# Effect of Distributing an Evidence-Based Guideline for Prevention of Osteoporosis on Health Education Programs in Municipal Health Centers: A Randomized Controlled Trial

**DOI:** 10.2188/jea.JE20110036

**Published:** 2012-03-05

**Authors:** Yoshimi Nakatani, Junko Tamaki, Misa Komatsu, Masayuki Iki, Etsuko Kajita

**Affiliations:** 1 Faculty of Nursing and Social Welfare Science, Fukui Prefectural University, Eiheiji, Japan; 2 Department of Public Health, Kinki University Faculty of Medicine, Osaka-sayama, Japan; 3 Seijyuji Nursing College, Komono, Japan; 4 Department of Public Health and Home Nursing, Nagoya University School of Health Sciences, Nagoya, Japan

**Keywords:** evidence-based guideline, fracture, health education, osteoporosis, randomized controlled trial

## Abstract

**Background:**

Current health education programs for osteoporosis prevention are not strictly evidence-based. We assessed whether distribution of an evidence-based guideline improved such programs at municipal health centers.

**Methods:**

This randomized controlled trial evaluated 100 municipal health centers throughout Japan that were randomly selected from those that planned to revise osteoporosis prevention programs. The implementation status of educational items recommended by the guideline was assessed before and after the intervention by evaluators blinded to the allocation. After the pre-intervention assessment, centers were randomly allocated in a 1:1 ratio to intervention and control groups by a minimization method defining region and city/town as stratification factors. Centers in the intervention group were given copies of the guideline; centers in the control group were instructed to use any information except the guideline. Analyses were performed on an intention-to-treat basis.

**Results:**

The guideline was used by 50% of the intervention group. Before the intervention, there was no significant difference in the evidence-based status of health education between the groups. The post-intervention assessment showed that the implementation rates of health education on dietary calcium intake for postmenopausal women and exercise for elderly persons were higher in the intervention group. Specific advice on intakes of calcium and vitamin D and exercise became more evidence-based in the intervention group.

**Conclusions:**

The findings suggest that the guideline helped healthcare professionals to improve health education programs by making them more evidence-based. However, the improvements seemed to be limited to items that the professionals felt prepared to improve.

## INTRODUCTION

Osteoporotic fracture is one of the greatest threats to the health and quality of life of elderly adults. Hip fracture is the most serious osteoporotic fracture, as about 10% of patients with this fracture die within 1 year and about 30% of them experience a decrease in activities of daily living as compared with prefracture levels.^1^ The proportion of hip fractures in Asia is expected to increase from a quarter of the world’s total in 1990 to about half by 2050 as a result of the aging of the population in that region.^2^^,^^3^ Therefore, the World Health Organization ranks osteoporosis as one of the most important health issues among noninfectious diseases.^4^

With recent progress in and dissemination of evidence-based medicine in clinical settings, the implementation of evidence-based preventive measures for important diseases such as osteoporosis is also needed in the field of community health. However, preventive programs for osteoporosis and osteoporotic fractures that are conducted by municipalities are not always evidence-based.^5^ Guidelines that convey evidence-based measures are expected to improve this situation. In Western countries, clinical practice guidelines for the prevention, diagnosis, and treatment of osteoporosis and osteoporotic fractures have been published to promote appropriate diagnostic procedures for the early detection and treatment of osteoporosis.^6^^–^^10^ High compliance with such guidelines by physicians has been reported to improve patient bone mineral density,^11^^–^^14^ reduce the number of days spent in hospital, and lower the cost of treatment.^15^^–^^17^ However, these studies were conducted mostly in clinical settings. Whether guidelines for osteoporosis prevention improve preventive activities by nurses, public health nurses, and allied health professionals in the field of community health has not been thoroughly investigated and has never been tested by rigorous evaluation such as in a randomized controlled trial (RCT).^18^^,^^19^

The effectiveness of guidelines is best analyzed by assessing disease reduction as an outcome, but such verification requires considerable time. Therefore, we used a surrogate outcome, namely, increase in the evidence-based status of preventive programs. Using this surrogate outcome, we conducted an RCT to assess whether municipal health centers that received an evidence-based guideline for the prevention of osteoporosis had a higher implementation rate for health education items on osteoporosis prevention recommended by the guideline.

## METHODS

### Study design

This was a randomized, controlled, parallel-group comparison trial conducted as a single-center study, with evaluators blinded to the allocation.

### Subject institutions

**Figure 1.**
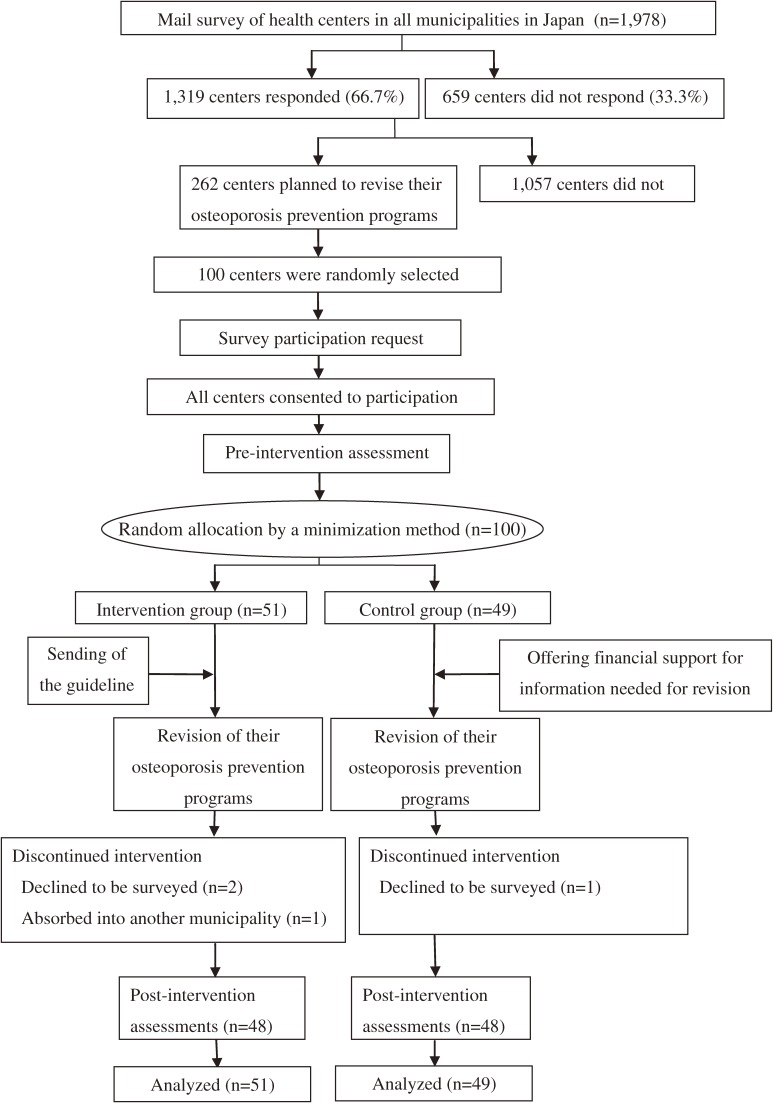
Flow diagram of the study

The analyzed institutions in the present study were 100 municipal health centers randomly selected from 262 centers that had indicated their intention to revise their osteoporosis prevention programs in a mail survey of 1978 municipalities throughout Japan conducted during November 2006 and January 2007, just before the present study (Figure 1).^5^ We explained the study protocol in writing, including the objectives of the study, study design, random allocation to the intervention or control group, content of the intervention, outcome measures, concealment of the allocation to evaluators, and study time line, to each of the selected institutions, which was followed by additional explanations by telephone. We obtained agreement from each institution regarding its participation in the study before conducting the pre-intervention assessment.

### Intervention

The intervention was the distribution of an evidence-based guideline. The guideline was entitled *“Evidence-Based Guideline for the Prevention of Osteoporosis and Osteoporotic Fractures in Community Health”*, a purely evidence-based practice guideline written in Japanese for the prevention of osteoporosis published in October 2004.^20^ This guideline was developed and formatted in accordance with recommendations for evidence-based guidelines, according to formal assessment procedures specified in the Japanese version of the AGREE instrument.^21^

The guideline^20^ evaluated all available evidence on potential preventive measures for osteoporosis prevention, including 8 areas of health education, namely, food intake, nutrient intake, exercise, exposure to sunlight, weight management, smoking and alcoholic beverage drinking control, and fall prevention. These measures targeted 3 groups, ie, premenopausal women (young women), postmenopausal women younger than 65 years (postmenopausal women), and men and women 65 years or older (elderly persons). The guideline presents a summary of the best available evidence for each of the health education items and classifies each item according to the summary of evidence into 5 grades of recommendation, as follows:

A: Implementation is strongly recommended based on sufficient evidence
B: Implementation is recommended based on evidence
C1: Implementation is appropriate but does not have sufficient scientific support
C2: Implementation is not recommended, due to lack of scientific support
D: Implementation is discouraged based on evidence

After random allocation of the health centers, the controller distributed free copies of the guideline to the intervention group and asked them (in writing) to use it to revise preventive programs for osteoporosis and osteoporotic fractures. In contrast, the controller instructed the control group (in writing) to use any information other than the guideline to revise the programs and offered to reimburse centers for the cost of materials such as manuals or books needed for this revision, in place of free distribution of the guideline.

### Outcome measures

The principal outcome was the implementation rate among municipal health centers for health education items on osteoporosis prevention recommended by the guideline. The secondary outcome was change in the evidence-based status of implemented advice for each health education item during the intervention period.

### Randomization

After the pre-intervention assessment, the 100 centers were randomly allocated in a 1:1 ratio to the intervention and control group by a minimization method that defined region and city/town as stratification factors. The allocation was performed by the controller of the trial (M. I.), who was not involved in the assessment as an evaluator.

### Survey items and assessment

Among the preventive measures evaluated by the guideline, we investigated the implementation status of 21 items in the health education program, for which the guideline^20^ gave a grade of recommendation of C1 or higher. These items were evaluated separately for the 3 target groups for osteoporosis prevention.

We asked whether the municipal health centers offered their residents each of the 21 items in their health education program for osteoporosis prevention. If they did, we assessed the extent to which health education was conducted based on evidence, before random allocation and after the intervention. These assessments were conducted by a registered nurse or licensed public health nurse, using a structured interview of personnel responsible for the osteoporosis prevention programs at the municipal centers. The post-intervention assessment was performed 1 year after the distribution of the guideline under blinded conditions in which the evaluators were unaware of the allocation.

### Statistical analysis

The principal comparisons of outcome measures between the intervention group and control group were performed on an intention-to-treat (ITT) basis, where the outcome measures of the municipal centers lost to follow-up were treated as unchanged during the intervention period. The principal outcome was the implementation rate for health education items on osteoporosis prevention recommended by the guideline, as measured in post-intervention assessments. We classified health education as implemented if the implemented advice attained the specific advice recommended by the guideline (eg, recommended dietary calcium intake was 800 mg/day). Each instance of implemented advice was assigned a score of 2, 1, 0, or −1 according to the grade of recommendation (A or B, C1, C2, or D, respectively) that the advice had attained. The sum of the scores for all implemented advice was calculated to yield an overall index of the evidence-based status of health education implemented in each health center. This score theoretically ranged from −3 to 41 and was not normally distributed. The difference in scores for the intervention and control groups was analyzed by the Mann-Whitney U-test before or after the intervention. We then performed the χ^2^ test or Fisher’s exact test to compare the implementation rates for each health education item between the intervention and control groups in the pre-intervention and post-intervention assessments.

The secondary outcome was change in the implemented advice for each health education item during the intervention period, which was classified into 3 categories: improved, unchanged, and worsened. We classified health education as improved if the implemented advice approximated the advice recommended by the guideline (eg, a dietary calcium intake of 200 to 600 mg/day) and worsened if advice was not consistent with the guideline. We assigned a score of 1, 0, or −1 to an improved, unchanged, or worsened health education item, respectively, to represent the change in the evidence-based status of each item. The sum of all the scores was used to represent overall change in the health education program that was implemented in the health center. The difference in scores for the intervention and control groups was tested by the Mann-Whitney U-test, with an adjustment for the number of ties.

In addition to the principal ITT analyses, we also conducted a per-protocol dataset (PPS) analysis in which the dataset included the health centers that completed the trial and those among the intervention group that reported using the guideline. Statistical analyses were performed with SPSS (version 15.0J; SPSS, Tokyo, Japan).

## RESULTS

### Characteristics of the health centers studied

All 100 municipal health centers completed the pre-intervention assessment. Of these, 3 centers declined to participate in the trial and 1 center was absorbed into another municipality (Figure 1). We performed the post-intervention assessments for the remaining 96 centers (48 in the intervention group and 48 in the control group; 96% follow-up rate). There were no significant differences between the intervention and control groups in municipality type, population, population aging rate, number of permanent health center staff, or the qualifications of the staff (physicians, public health nurses, nurses, dieticians, physical therapists, and clerks). There was no significant difference between the intervention and control groups in the implementation rate for osteoporosis screening or any type of health education or counseling before the intervention.

### Reference materials used for revising health education programs

**Figure 2.**
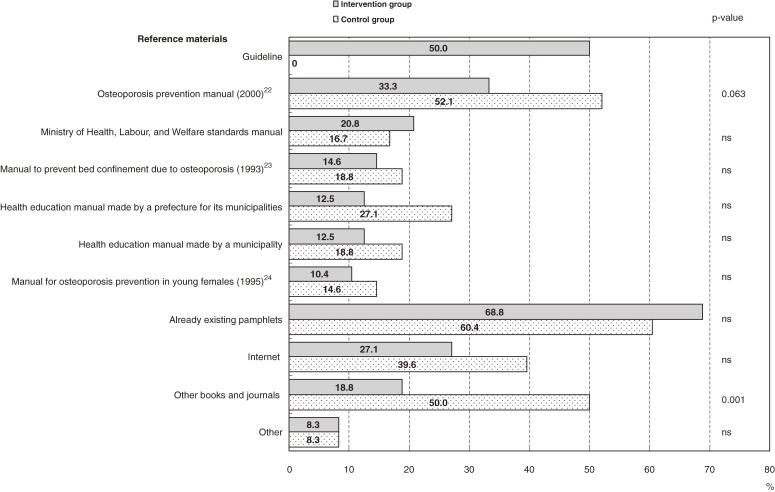
Reference materials used to revise health education programs for osteoporosis prevention in the intervention and control groups. Use of multiple reference materials was allowed. *P*-values were calculated by the χ^2^ test. ns: not significant.

As a result of the intervention, 50% of the health centers in the intervention group and none in the control group used the guideline as reference material when they revised their osteoporosis prevention programs. Pre-existing booklets or manuals^22^^–^^24^ were the most frequently used reference material in both the control intervention groups (Figure 2). Three centers in the control group sought and received reimbursement for information materials needed for the revision.

### Pre-intervention status of implementation of evidence-based health education

There was no significant difference in the overall score for the implementation status of evidence-based health education items, as recommended by the guideline, between the intervention (median, 10; first and third quartiles: 3, 17) and control (median, 9; first and third quartiles: 1.5, 18.5) groups in the pre-intervention assessment. The Table shows the implementation status of each health education item in these groups. Sufficient intakes of milk/dairy products and calcium and brisk walking and fall prevention were relatively frequently implemented, as recommended by the guideline, but most of the other items were not. There was no difference in the implementation rate for any health education item between the 2 groups.

**Table.** Frequency of implementation and implementation rate of evidence-based health education items for osteoporosis prevention, as recommended by the guideline, in the intervention and control groups before and after the intervention

Health education items	Specific advice recommended by the guideline	Target populations^b^ (Grade of recommendation)	Pre-intervention^a^			Post-intervention^a^			
	
			Intervention group (*n* = 51)	Control group (*n* = 49)	*P* for group difference^c^	Intervention group (*n* = 51)	Control group (*n* = 49)	*P* for group difference^c^	*P* for group difference^d^
Foods									
Milk and dairy products	1 cup/day	Young (C1)	4 (7.8%)	4 (8.2%)	ns	8 (15.7%)	6 (12.2%)	ns	ns
		Post (B)	16 (31.4%)	19 (38.8%)	ns	26 (51.0%)	20 (40.8%)	ns	0.046
		Elderly (C1)	16 (31.4%)	18 (36.7%)	ns	23 (45.1%)	20 (40.8%)	ns	0.095
Soy products	Habitual consumption	Young (C1)	19 (37.3%)	14 (28.6%)	ns	27 (52.9%)	20 (40.8%)	ns	0.095
		Post (B)	20 (39.2%)	16 (32.7%)	ns	28 (54.9%)	20 (40.8%)	ns	0.046
		Elderly (C1)	19 (37.3%)	16 (32.7%)	ns	26 (51.0%)	20 (40.8%)	ns	0.095
Nutrients									
Calcium intake	At least 800 mg/day from diet	Young (B)	22 (43.1%)	25 (51.0%)	ns	29 (56.9%)	22 (44.9%)	ns	0.095
		Post (A)	23 (45.1%)	26 (53.1%)	ns	33 (64.7%)	23 (46.9%)	0.074	0.010
		Elderly men (C1) women (B)	22 (43.1%)	23 (46.9%)	ns	29 (56.9%)	21 (42.9%)	ns	0.030
Calcium supplement	1000 mg/day	Young (A)	0	0	ns	1 (2.0%)	0	ns	ns
		Post (B)	0	0	ns	2 (3.9%)	0	ns	ns
		Elderly men (C1) women (A)	0	0	ns	2 (3.9%)	0	ns	ns
Vitamin D intake	10 µg/day	Young (C1)	1 (2.0%)	0	ns	2 (3.9%)	0	ns	ns
	10 µg/day	Post (B)	1 (2.0%)	0	ns	2 (3.9%)	0	ns	ns
	20 µg/day	Elderly (B)	0	0	ns	1 (2.0%)	0	ns	ns
Magnesium intake	300 mg/day	Young (C1)	1 (2.0%)	1 (2.0%)	ns	2 (3.9%)	0	ns	ns
	100–300 mg/day	Post (B)	1 (2.0%)	0	ns	2 (3.9%)	0	ns	ns
	Less than 300 mg/day	Elderly (C1)	0	0	ns	1 (2.0%)	0	ns	ns
Isoflavone intake	40–50 mg/day	Young (C1)	3 (5.9%)	2 (4.1%)	ns	5 (9.8%)	4 (8.2%)	ns	ns
		Post (B)	3 (5.9%)	2 (4.1%)	ns	8 (15.7%)	5 (10.2%)	ns	ns
Exercise									
Brisk walking	30 min/day at least 3 time a week	Elderly (C1)	19 (37.3%)	14 (28.6%)	ns	25 (49.0%)	10 (20.4%)	0.003	0.028
High-impact training	Habitual exercise	Young (B)	2 (3.9%)	2 (4.1%)	ns	10 (19.6%)	4 (8.2%)	ns	ns
	Habitual exercise	Post (A)	2 (3.9%)	2 (4.1%)	ns	9 (17.6%)	5 (10.2%)	ns	ns
	Exercise that can be continued safely	Elderly (B)	2 (3.9%)	2 (4.1%)	ns	11 (21.6%)	5 (10.2%)	ns	ns
Low-impact training	15 min/day at least 3 time a week	Elderly (C1)	8 (15.7%)	4 (8.2%)	ns	12 (23.5%)	2 (4.1%)	0.005	0.014
Being active in everyday life	More active in everyday life	Elderly (A)	1 (2.0%)	0	ns	2 (3.9%)	2 (4.1%)	ns	ns
Strengthening of back muscles	15 min/day at least 3 time a week	Elderly (C1)	2 (3.9%)	0	ns	3 (5.9%)	1 (2.0%)	ns	ns
Exposure to sunlight	Inadvisable	Young (D)	4 (7.8%)	6 (12.2%)	ns	2 (3.9%)	5 (10.2%)	ns	ns
		Post (D)	4 (7.8%)	6 (12.2%)	ns	2 (3.9%)	4 (8.2%)	ns	ns
		Elderly (D)	4 (7.8%)	5 (10.2%)	ns	2 (3.9%)	4 (8.2%)	ns	ns
Weight management									
Maintenance of appropriate weight	Suggesting a desirable weight	Young (C1)	15 (29.4%)	8 (16.3%)	ns	12 (23.5%)	12 (24.5%)	ns	ns
		Post (B)	14 (27.5%)	8 (16.3%)	ns	12 (23.5%)	12 (24.5%)	ns	ns
		Elderly (B)	13 (25.5%)	7 (14.3%)	ns	10 (19.6%)	11 (22.4%)	ns	ns
Smoking and drinking									
Do not start smoking	Not to smoke	Young (B)	9 (17.6%)	8 (16.3%)	ns	3 (5.9%)	6 (12.2%)	ns	ns
		Post (A)	8 (15.7%)	8 (16.3%)	ns	4 (7.8%)	6 (12.2%)	ns	ns
Stop smoking	Strongly advised to quit smoking	Young (A)	6 (11.8%)	5 (10.2%)	ns	4 (7.8%)	2 (4.1%)	ns	ns
		Post (A)	5 (9.8%)	5 (10.2%)	ns	3 (5.9%)	1 (2.0%)	ns	ns
		Elderly (A)	5 (9.8%)	5 (10.2%)	ns	3 (5.9%)	1 (2.0%)	ns	ns
Alcohol drinking	Alcohol consumption less than 25 g/day	Elderly (C1)	11 (21.6%)	7 (14.3%)	ns	10 (19.6%)	8 (16.3%)	ns	ns
Fall prevention									
For elderly subjects with a ​ history of falls	Concrete advice	Elderly (C1)	24 (47.1%)	30 (61.2%)	ns	23 (45.1%)	19 (38.8%)	ns	ns
Total body exercise including ​ balance	At least 3 time a week	Post (A)	8 (15.7%)	10 (20.4%)	ns	8 (15.7%)	5 (10.2%)	ns	ns
		Elderly (A)	11 (21.6%)	15 (30.6%)	ns	13 (25.5%)	8 (16.3%)	ns	ns
Modification of behavior after ​ examination of risk factors	Concrete advice	Post (B)	15 (29.4%)	15 (30.6%)	ns	10 (19.6%)	8 (16.3%)	ns	ns
		Elderly (B)	22 (43.1%)	20 (40.8%)	ns	18 (35.3%)	13 (26.5%)	ns	ns
Environmental improvement	Concrete advice	Post (B)	17 (33.3%)	14 (28.6%)	ns	10 (19.6%)	8 (16.3%)	ns	ns
		Elderly (B)	26 (51.0%)	20 (40.8%)	ns	19 (37.3%)	14 (28.6%)	ns	ns

^a^Values show the number of health centers where the health education item recommended by the guideline was implemented, with the proportion of such centers to the number of centers in each group in parentheses.

^b^Young: premenopausal women, Post: postmenopausal women younger than 65 years, Elderly: adults 65 years or older.

^c^Evaluated by χ^2^ test or Fisher’s exact test on an intention-to-treat basis.

^d^Evaluated by χ^2^ test or Fisher’s exact test on a per-protocol dataset basis.

ns: not significant.

### Post-intervention status of implementation of evidence-based health education

The overall score for the evidence-based status of health education after the intervention was significantly higher (*P* = 0.045) in the intervention group (median, 12; first and third quartiles: 7, 18) than in the control group (median, 8; first and third quartiles: 1, 15.5) in the ITT analysis.

The Table also shows the implementation status for each educational item in both groups in the ITT analysis. Some items regarding food intake, including daily intake of at least 1 cup per day and habitual consumption of soybean products, were observed more often in the intervention group than in the control group, but these differences were not statistically significant. Regarding nutrient intakes, consumption of at least 800 mg/day of dietary calcium was more likely (*P* = 0.074) to be implemented in the intervention group than in the control group. The implementation rate for nutrient advice specifying precise intakes was low after the intervention and did not differ between groups.

Regarding exercise items, brisk walking and low-impact training, such as stretching in elderly persons, were used significantly more often in the intervention group than in the control group (*P* < 0.01). Performing high-impact training was observed more often in the intervention group than in the control group, but the difference did not reach statistical significance.

We found no significant difference between the groups in the frequency of implementing advice on exposure to sunlight, weight management, smoking, or fall prevention into health education programs.

In the PPS analysis, the intervention group (*n* = 24) had a significantly higher overall score for evidence-based status (*P* = 0.012) (median, 13.5; first and third quartiles: 8, 23.3) than did the control group (*n* = 48) (median, 8.5; first and third quartiles: 1.5, 15.8). In comparisons of implementation rates for health education items (far-right column of Table), the number of items that had a significantly or almost significantly higher rate (*P* < 0.1) in the intervention group increased from 3 items in the ITT analysis to 5 items (milk/dairy products and soy products plus the 3 items in the ITT analysis).

### Change during the intervention in evidence-based status of health education items

**Figure 3.**
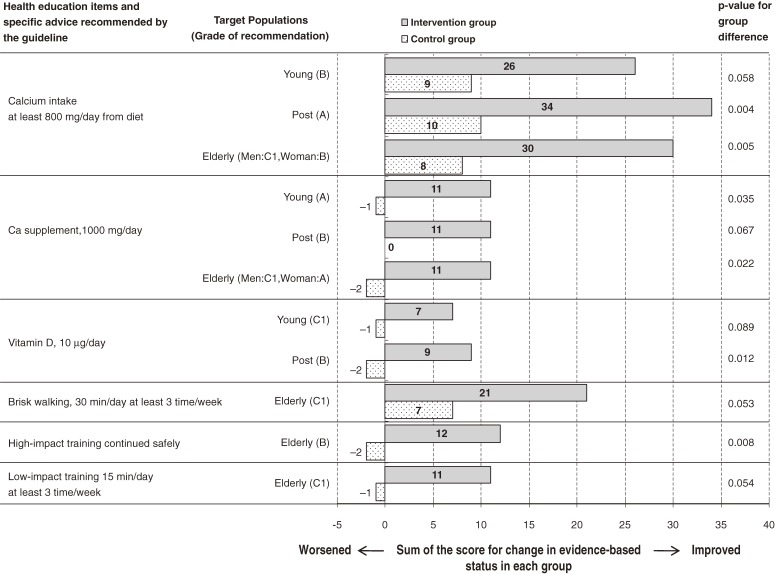
Comparison of scores for changes in evidence-based status of health education items between the intervention and control groups. The score was determined by assigning 1, 0, or −1 to improved, unchanged, or worsened evidence-based status of each health education item, respectively, during the intervention period. The horizontal axis denotes the sum of the scores in the intervention and control groups. Young: premenopausal women, Post: postmenopausal women younger than 65 years, Elderly: adults 65 years or older. *P*-values were calculated by the Mann-Whitney U-test.

We compared the overall score for changes in the evidence-based status of educational items during the intervention period between the intervention and control groups in the ITT analysis and observed that educational items became significantly more evidence-based (*P* = 0.050) in the intervention group (median, 10; first and third quartiles: 2, 19) than in the control group (median, 6; first and third quartiles: −5, 13.5). Figure 3 shows the items with significant or nearly significant differences (*P* < 0.1) in the change in evidence-based status between these groups. Educational items including calcium intake from diet or from supplements, vitamin D intake, and high- and low-impact exercise were more likely to have improved in the intervention group than in the control group. However, there was no significant difference between groups in the change in evidence-based status for education on sunbathing, weight management, smoking and drinking habits, and fall prevention (data not shown).

In the PPS analysis, the intervention group showed significantly greater overall improvement in the evidence-based status of health education (*P* = 0.013) (median, 12; first and third quartiles: 7.3, 21.5) as compared with the control group (median, 6; first and third quartiles: −0.8, 13.8). The number of items with significant or nearly significant differences (*P* < 0.1) in the change in evidence-based status between the groups increased by 6 items, as compared with the ITT analysis, which included milk/dairy products, soy products, magnesium intake, isoflavone intake, inadvisability of sun bathing, and fall prevention.

## DISCUSSION

This relatively small nationwide RCT of the effectiveness of distributing an evidence-based guideline for osteoporosis prevention to municipal health centers showed that the guideline may have helped healthcare professionals in improving the evidence-based status of several items in their health education programs. To the best of our knowledge, this is the first study to examine the effectiveness of an evidence-based guideline for health education in the field of community health. Evidence-based practice guidelines are one of the most promising tools for communicating the latest available evidence to practitioners. The effectiveness of evidence-based guidelines must be supported by evidence, such as that provided for the first time by the present study.

The intervention tested in this study was quite simple: we distributed a guideline by mail. However, even if a guideline is distributed, it is not always used. In fact, only half of the health centers in the intervention group used the present guideline. This rate of use was not satisfactory but was at least as high as rates reported in previous studies, which ranged from 35% to 49%.^25^^–^^27^ Because the extent of compliance with guideline use should be included in the evaluation of the performance of guideline, we analyzed its effectiveness on an ITT basis. When we compared the magnitude of improvement in evidence-based status between the results of the ITT and PPS analyses, the improvement was greater in the PPS analysis. This suggests that an increase in guideline use would further improve the evidence-based status of health education. No single method has been established to increase the use of a guideline; however, studies have indicated that it is necessary to increase awareness of the necessity of guidelines. In addition, healthcare professionals must acquire the knowledge necessary for implementing recommendations and seek education to increase skills and would benefit from the provision of information especially linked to performance, the development of tools to supplement the guideline, and management support.^28^^–^^33^

The effectiveness of the guideline varied by health education item. Intakes of calcium and vitamin D and exercise became more evidence-based in the intervention group than in the control group during the intervention period. However, there was no difference between groups in many of the other items on health education, even though relatively high levels of scientific evidence indicate that they are effective in preventing osteoporosis and fracture. It has been reported that guideline use is affected by prior recognition of the usefulness of the advice given by the guideline and by the presence of the knowledge and skills necessary for its implementation.^26^^,^^27^^,^^34^ Education on osteoporosis prevention by municipal health centers was legislated in 1990 in Japan, a country where calcium intake and habitual exercise have been ranked as important items for osteoporosis prevention. Health education skills for these items have been increasing during the 20 years of practice. Improvement in the evidence-based status of education in the present study thus appears to have occurred in the items that public health nurses and dieticians were professionally prepared to improve.

In addition to the preparedness of health professionals, systematic and environmental level factors, such as distribution of personnel, limitations in time and resources, and the awareness and attitudes of administrators or project managers have been reported to affect guideline compliance.^35^^,^^36^ Only 1 or 2 dieticians per municipality were typically available in the analyzed centers.^5^ Availability of adequate personnel for nutrition education was sometimes marginal, which may have hindered improvements in education on nutrient intake that were recommended by the guideline.

Education on fall prevention has long been conducted in municipal health centers in order to prevent elderly people from becoming bedridden due to fractures or serious injuries. However, the implementation rate of education for fall prevention was low both in the intervention and control groups, even after the intervention. As David et al^28^ suggested, laws and regulations often affect implementation of preventive measures recommended by guidelines. This could have been the case in the present study. A revision of the Long-Term Care Insurance Law, which transferred fall prevention programs from health centers to nursing care insurance divisions in municipalities, came into force in 2006,^37^ when the intervention was implemented. Unfortunately, we were unable to evaluate the preventive activities of municipal divisions other than health centers in the present study.

The present study has several strengths. The analyzed health centers were randomly selected throughout Japan, and the completion rates in the trial were optimal, which suggests limited selection bias. The study employed an RCT design with evaluators being blinded to allocation, which eliminates a range of biases.

Limitations of the study should also be noted. The study did not use a double-blind design because it was not possible to use a placebo guideline. Instead, we offered to reimburse the control centers for the cost for materials needed to revise their health education programs. Although only 3 centers claimed reimbursement, our offer may have increased the use of information other than the guideline in the control group and may have improved the evidence-based status of the programs of the control centers, thereby decreasing the magnitude of differences in the outcome measures between the groups. The intervention was simply the distribution of a guideline, without any supplemental tools or services to improve compliance. This resulted in 50% compliance for guideline use. Although not lower than rates in previous studies,^25^^–^^27^ this rate was nevertheless unsatisfactory. The intervention time was 1 year, which may not have been long enough to revise health education programs. All differences in implementation rates of health educational items between the intervention and control groups were tested independently. This multi-hypothesis testing may have increased the probability of a Type I error, and thus the results should be interpreted with caution. The process by which the intervention led to change in the outcome (ie, whether health professionals actually used the guideline, whether they understood the content of the guideline, and whether they agreed with the recommendations provided by the guideline) was not evaluated in detail. The outcome measures were not changes in patient or client factors, such as change in lifestyle or behavior, increase in bone mineral density, or decrease in fracture rate. The sample size was small, resulting in low statistical power. Finally, we had no control over legislation passed by the Japanese government during the study.

These limitations suggest agenda items for future studies. Detailed assessment of the process from intervention to change in outcome would reinforce the effectiveness of the guideline and might provide insight on how to improve guideline compliance. This would include supplemental tools to increase awareness of the importance of the guideline, knowledge to implement countermeasures recommended in the guideline, and skills to educate clients in the manner recommended by the guideline. The effectiveness of such practical interventions should also be tested by large-scale RCTs with an intervention period long enough to complete revisions to the program.

In conclusion, the findings of the present RCT suggest that distribution of a guideline helped healthcare professionals in municipal health centers improve the evidence-based status of several items in health education programs for osteoporosis prevention. However, only half of the intervention group used the guideline, and the health education items that improved were those which healthcare professionals felt sufficiently prepared to implement. Therefore, the guideline should be accompanied with supplemental tools to increase compliance with guideline use.
